# Developing a behaviour identification method for healthcare waste management: a case study of hospital waste management in Australia

**DOI:** 10.1186/s12913-025-13463-5

**Published:** 2025-10-02

**Authors:** Lena Jungbluth, Janette Wright, Peter Bragge, Denise Goodwin

**Affiliations:** 1 Monash Sustainable Development Institute, BehaviourWorks Australia, Monash University, 8 Scenic Boulevard, Clayton, VIC 3800 Australia; 2 Royal Melbourne Hospital, 300 Grattan Street, Parkville, VIC 3050 Australia; 3 Monash Sustainable Development Institute Evidence Review Service, BehaviourWorks Australia, 8 Scenic Boulevard, Clayton, VIC 3800 Australia

**Keywords:** Healthcare, Waste management, Behaviour change, Behaviour identification, Waste flow diagrams

## Abstract

**Background:**

Healthcare generates millions of tonnes of waste each year, with some of this waste not getting disposed of in its most appropriate streams. Due to the importance of correct sorting and transporting of generated waste, human behaviour plays a crucial role in the waste disposal process. Thus taking a behavioural science approach to the challenge of healthcare waste management has potential to value-add to existing research and practice efforts. A fundamental step in applied behaviour change projects is the identification of specific behaviours that can have positive impact. To support this, this project developed a new method to comprehensively identify and map the often interconnected waste management behaviours in a healthcare setting.

**Methods:**

To develop the new behaviour identification method, previous work on ‘waste flow diagrams’ was extended and overlaid with principles of behavioural science, clearly defining the identified behaviours with regards to their actor, action, target, context and time. The method was applied to the management of waste generated in the perioperative area in a large, metropolitan, public hospital in Australia.

**Results:**

Applying the developed method to the management of waste from the described hospital setting identified 184 specific, interconnected waste management behaviours performed by thirteen actor groups (including anaesthetists, surgeons, nurses, technicians, cleaning staff).

**Conclusion:**

This project has applied established principles of behavioural science to traditional waste flow diagrams and thereby developed a new behaviour identification method. The developed method lays the groundwork for future behaviour prioritisation work, which can be followed by an investigation of influences on behaviour performance. Ultimately, this process can support the design of a targeted behaviour change intervention to improve healthcare waste management.

**Supplementary Information:**

The online version contains supplementary material available at 10.1186/s12913-025-13463-5.

## Background

Healthcare generates millions of tonnes of waste each year [1]. In the year 2022/2023, the UK National Health Service in England for example created 473,400 tonnes of waste [2], while healthcare and its supply chain accounted for 8% of waste in the Australian state of New South Wales in 2017 [3]. The World Health Organization estimates that around 15% of healthcare waste is hazardous, requiring special treatment, such as incineration, as part of the disposal process [4, 5]. The disposal of hazardous healthcare waste is costly [6] and environmentally damaging [7, 8]. The remaining 85% of healthcare waste is estimated to be non-hazardous allowing for disposal as general waste [5]. Where local laws and regulations allow to do so, some of this waste can even be recycled [9], thereby reducing its environmental impact [10].

While there is ongoing exploration into ways to use digital technologies, such as artificial intelligence and robotics, to support or automate medical waste management [11], the current state of healthcare waste management still requires many manual tasks [12]. Hence, human behaviour plays a crucial role in the disposal of waste in the most suitable manner, because items need to be placed into their appropriate waste stream during the initial disposal process [10] and handled accordingly during subsequent transport [13]. Studies show that this is not always the case: For example, hazardous waste can be found in non-hazardous waste streams [14], non-hazardous waste gets placed into hazardous waste streams, or recycling opportunities get missed by items not being placed in their appropriate recycling stream [15–17]. Reasons for these misclassifications can be manifold, including a lack of appropriate bins [18] or a lack of knowledge on or interest in correct waste disposal [14]. This suggests that there is room for improvement with regards to waste management behaviour, which, if done appropriately, results in a safer, less costly and less environmentally impactful waste disposal of healthcare waste [9].

### Behavioural science to design behaviour change interventions

Interventions to change human behaviour can be designed by drawing on behavioural science [19]. According to Hagger et al. (2020) [20], commonly used approaches to design behaviour change interventions include, among others, the Behaviour Change Wheel [19], the four step approach by French et al. (2012) drawing on the Theoretical Domains Framework [21], or the Intervention Mapping Approach [22].

These approaches are topic-agnostic and can be used for any issue where human behaviour plays a role, because the steps inherent in their intervention design process take into account topic-specific and contextually relevant aspects [19]. They cover largely similar steps (or a subsection of thereof), in that they start off with understanding the problem and context of interest, identify target audiences and behaviours, perform a theory-informed investigation into facilitators and barriers to behaviour performance and then design an intervention to reduce identified barriers and strengthen identified facilitators. This is followed by the final steps of implementing, testing and evaluating the intervention (for a similar comparison see also Hagger et al. (2020) [20] and Presseau et al. (2021) [23]). What early stages of these approaches have in common is a strong focus on expressing the problem in ‘behavioural terms’. This means they clearly articulate which audiences need to perform which specific behaviours, or ‘who needs to do what, differently’ [21, p. 2], for the problem to be improved [23].

*Clearly* expressing behaviours of interest lays the foundation for a targeted investigation into their specific facilitators and barriers [24, 25]. Such a targeted investigation ensures that assumptions are avoided and instead topic-specific and contextually relevant influences on behavioural performance are understood, before the behaviour change intervention is designed [19]. Letting the subsequent intervention design be informed by the obtained insights on what hinders or helps with the performance of the behaviour(s) in question, increases the likelihood of success of the designed intervention [19]. However, facilitators and barriers to behavioural performance can differ even between seemingly similar behaviours [26]. Therefore a *clear* definition of the behaviours in question lays the foundation for the success of the subsequently following steps in the intervention design process [25].

A clearly articulated behaviour includes the following components: an actor, an action, a target, a context and a time [25, 27]. Actors in healthcare waste management can include, for example, nurses, doctors and cleaners. Actions describe the disposal process (e.g. ‘to place in a bin’). The target specifies ‘what’ is placed in a bin (e.g. ‘clinical waste items’, ‘general waste items’, etc.) and can differ depending on the different waste streams that occur in a given setting. The context refers to the area within a healthcare setting where a behaviour is performed, e.g. a hospital ward. In cases where contextual differences exist between individual rooms or areas within one ward, this specification might be as nuanced as the room-level. The time component can simply read as ‘always’ or be more specific, like ‘before/ during/ after surgery’. Following this, a clearly expressed waste management behaviour, for example, reads ‘Cleaners place an empty clinical waste bin in the dirty utility room of the hospital’s perioperative area overnight’.

Expressing behaviours in a detailed manner can result in long lists of behaviours different actors can perform to address an issue. For example, a program in Australia identified through a literature review and input from experts, 241 behaviours householders could undertake to improve their energy use [28]. Such long lists of behaviours can usually not be addressed all at once in one intervention [29]. Thus, prioritising behaviours to target the most important ones in an intervention is a recommended approach [19, 30, 31]. However, in order to perform a meaningful prioritisation exercise, it is important to start with an as comprehensive pool of behaviours to prioritise from as possible [29]. No known study so far has developed a method to comprehensively identify behaviours involved in the waste management in a healthcare setting, which presents a gap in the current literature.

### Creating maps to improve healthcare waste management

The creation of maps is used in healthcare improvement projects [32] and represents one way of identifying behaviours to improve a problem at hand [29]. Additionally, mapping behaviours shows how they influence each other and thereby accounts for their interconnectedness [19, 33]. This is very relevant in the context of healthcare waste management, where staff performing waste sorting behaviours rely on waste support staff to prepare and empty bins [34] as well as to dispose of waste appropriately throughout the healthcare waste management system [13].

A number of maps concerning the topic of healthcare waste or material management have been developed using different methodologies. These methodologies include systems dynamics, mapping out the interconnectedness of various systemic factors related to healthcare waste management [35–37]. However, due to their focus on the systemic nature of healthcare waste management, their primary purpose does not lie on identifying behaviours in the level of detail of ‘who needs to do what, differently’ [21, p. 2] in a given context. Another methodology that has been applied to healthcare material management, and whose results are recommended to be depicted in a map [38], is material flow analysis. This methodology undertakes ‘a systematic assessment of the flows and stocks of materials within a system defined in space and time’ [38, p. 3]. Studies in the healthcare context using this approach include maps showing how different materials, such as medication and plastics, flow in and out of a given healthcare location [39–41]. However, this methodology has an explicit focus on materials and has not been designed to include actors and their behaviours.

The most promising mapping approach for behaviour identification that could be identified in the existing literature consisted of a mapping method resulting in ‘waste flow diagrams’ (WFDs) [42–44]. This method has been developed and applied to describe the waste management in healthcare settings [42–44]. The method is derived from Structured Systems Analysis and Design Method (SSADM), which includes data flow diagrams [42–44]. SSADM and its data flow diagrams originate from the field of information systems and have clear notation conventions for depicting the flow of information through the system [43, 45]. The authors of the mapping method to create WFDs [42–44] saw a parallel between data and waste flowing through a system and hence adapted the notation conventions of data flow diagrams for, what they termed, ‘waste flow diagrams’. Due to the granularity with which WFDs describe waste management steps, this method presents a promising starting point to identify behaviours involved in the healthcare waste management process. However, like the previous mapping examples, this mapping method has also not been designed with an explicit behavioural lens; that is, it does not aim to identify and describe waste management behaviours in terms of actors, actions, targets, context and time [25, 27].

Drawing on the aspects outlined above, this paper addresses the gap that so far no method to comprehensively identify waste management behaviours in a healthcare setting has been developed. Specifically, this paper aims to develop and test a new method to comprehensively identify and map the often interconnected waste management behaviours in a healthcare setting by expanding on an existing healthcare waste mapping approach.

## Methods

### The behaviour identification method

In order to develop the behaviour identification method, the method to create WFDs [42–44] was adapted. The key innovation in this iteration of waste mapping is the overlaying of described behaviours using established principles of behavioural science. Table 1 outlines the notation conventions for WFDs as set by their authors [42–44], as well as how the notation conventions were refined for the use in the behaviour identification method. Since overall WFDs can contain a lot of information, the behaviour identification method adds some layout rules to facilitate ease of reading a created map: A map should be structured according to different locations within a given healthcare site, aiming for a flow from top to bottom. This means that the top of the map shows the locations of waste generation, i.e. where materials turn into waste. Each location should indicate which waste materials get created there. The level below shows bins in these locations. The following levels show one interim storage level each, with the final storage location at the very bottom of the map (to better visualise this, see created waste map in Additional file 1).

Behaviours are added as appropriate for each level: In doing so, the top level shows all material disposal behaviours, the next level shows the waste removal behaviours from the location of waste generation. The following levels show interim waste removal and transport behaviours until the materials reach their final destination, e.g. in the waste dock.

**Table 1:** Comparison of notation rules

Notation rules for WFDs [42–44]	Notation rules for waste maps based on the behaviour identification method
Box to identify waste storage areas. IDs are unique identifiers consisting of a combination of letters and numbers (W#=bins/bags; T#=temporary storage area, e.g. on wards; M#=main storage area, e.g. waste dock; S#=permanent storage, e.g. for records).	Twofold use of this box: 1 (unlike WFDs) - To indicate the material once it has turned to waste, e.g. clinical waste, general waste, etc. Materials are identified by the letter ‘M’ and a running number which repeats if the same material occurs at multiple locations. The use of the letter ‘M’ is different from WFDs, which reserve it for main storage areas. 2 (like WFDs) - Waste storage areas. But unlike WFDs, no unique IDs are used for bins, instead a bin icon is used for ease of distinction between bins and locations (e.g. a room or area at a healthcare site). Storage locations are identified by the letter ‘S’ and a running number for each location at the healthcare site (i.e. multiple boxes can have the same ID if they are located in the same location). The use of the letter ‘S’ is different from WFDs, which reserve it to indicate permanent storage. Unique identifying numbers are of less importance in the behaviour identification method as it depicts the entire waste management process in one map and does not allow for the creation of multiple linked maps.
Process box to describe processes (usually activities) that ‘affect or change’ the waste [44, *p*. 1200]. It indicates either who performs the process or where it is performed. References are unique, sequentially allocated identifiers.	Behaviour box for a detailed description of waste management behaviours per waste stream using established principles of behavioural science. This means they include details on the actor, action, target and time where necessary (the context, i.e. where the behaviour occurs, can be omitted in this box since the location of this box in the map clarifies this). This box is similar to the WFDs’ process box but its behavioural focus is more explicit due to its explicit requirement of a detailed behavioural definition. Moreover, unlike WFDs, the top row is reserved solely for the actor(s) of the behaviour, while WFDs leave it open as to whether an actor or a location is described. Unlike WFDs, the reference is removed, as some behaviours could be performed by multiple actors. In such cases a behaviour box describes as many behaviours as there are actors listed. Hence a unique identifier would be misleading.
Directional arrows to describe the waste flow. Arrows are labelled and each waste stream receives its own arrow.	Like WFDs arrows indicate the waste flow but the behaviour identification method expresses different nuances. Directional colour-coded arrows indicate waste flow *as intended* (regardless of whether this is current practice). Dotted arrows are used if a recyclable material has to be disposed into a non-recycling stream because this stream does not exist at the healthcare site (a lack of opportunity [19, 46]). Unlike WFDs, the behaviour identification method does not capture undesirable waste management behaviours present in current practice. It only focusses on desirable behaviours, since behavioural science approaches recommend, where possible, to focus on behaviours that can improve the situation rather than on problem behaviours [24, 29]. This is recommended because desirable behaviours are more instructive than undesirable ones, as they inform a given target audience about what they *should* do, as opposed to telling them what they *should not* do without providing a suitable alternative [29]. Arrows are colour-coded according to the actor who performs the behaviour. If a behaviour box shows multiple actors, multiple colour-coded arrows need to be used. Due the level of detail provided with the material/waste storage box and the behaviour box, labelling of arrows is redundant.
External entity which is outside the study boundaries. Waste flows (i.e. arrows) cannot be linked directly from a waste storage area to an external entity.	Like WFDs, external entity which is outside the study boundaries. Unlike WFDs, waste flows (i.e. arrows) can be linked directly from a waste storage area to external entities if the waste management behaviour is performed by the external entity. This means, for example, if a waste contractor is emptying a bin in a truck, this behaviour is not identified via a behaviour box, if it is outside the system boundaries. Instead an arrow is drawn directly from the waste storage area to the external entity.
To reduce the crossing over of arrows, storage areas can be repeated in the same or additional diagrams. This is indicated by a second line in the ID field and the same unique identifiers in the diagrams.	To represent the interconnectedness of the waste management process, it was deemed important to keep all elements in the same map. This means that some crossing over of arrows is unavoidable. It also reduces the importance of unique identifiers as they are no longer necessary to identify the same items in various linked maps.

### Case study to apply the behaviour identification method

To demonstrate the usability of the behaviour identification method, it was applied to the management of waste generated in the perioperative area of the Royal Melbourne Hospital, which is a large, metropolitan, public hospital based in Melbourne, Australia. A perioperative area was chosen as case study site, as these locations are a substantial contributor to hospital waste [47]. Ethics and governance approval was obtained for all research activities from the hospital’s Human Research Ethics Committee (HREC/85075/MH-2022) and Office for Research respectively.

#### Observations

In August and September 2022, the first author performed 18 h of hospital observations across four days to understand the local waste-related behaviours and their context. Observations mainly focussed on a combination of the layout, set up and environmental context of the perioperative area as well as staff behaviour. For the latter, 34 staff were observed during 9 different cases (i.e. surgeries or parts thereof). Specific activities of interest were selected for observation, such as preparation for, or clean-up after surgeries. A brief visit to other hospital areas relevant to waste management, e.g. the waste dock and the sterile store, was also part of the observation activities. Verbal consent was sought from participants in line with the received ethics approval.

Observations were recorded in form of paper notes and photographs and were subsequently summarised electronically to capture overall reflections, notes on the physical setting and bins, as well as on staff behaviour. The insights from the observations were then used by the first author to create a basic draft waste map on paper and in the online platform Miro [48] in preparation for two behaviour identification workshops with selected hospital staff.

#### Behaviour identification workshops

In September 2022, two consecutive two-hour behaviour identification workshops were held, facilitated by the first author. The workshops were designed to result in a waste map, applying the developed behaviour identification method.

Both workshops were held in person at the partner hospital, but due to availability limitations, during both workshops one participant joined via the videoconferencing platform Zoom. The workshops were augmented by two individual one-on-one conversations (via Zoom and face-to-face) between the first author and specific participants, who were unable to join the workshops, but whose perspectives were considered important. The insights from each conversation were integrated with the group work from the workshops and are reflected in the final outputs.

Each of the two workshops (including the additional conversations) was attended by seven participants, involving nine individuals in total, who were all staff members of the partner hospital. The following perspectives were represented at one or both workshop/conversation sessions by one or multiple participants: Anaesthetist, anaesthetic nurse, surgical nurse, technician, environmental services and facilities management. All workshop and conversation sessions were audio recorded. Written consent to participation and recording was obtained from all participants.

During the first workshop, the basic draft waste map, that was created following the observations, was presented to workshop participants to check for accuracy and to expand on further based on their knowledge. To be efficient with workshop participants’ time, following the first workshop, the first author further developed the waste map and again presented the draft at the second workshop for participants to correct and complete.

The mapping exercise focussed on clinical locations within the perioperative area, where most waste was being created, as identified by the observations and discussions with workshop participants. It included the set up rooms (where instruments are being prepared for surgery), the anaesthetic bay (where patients receive anaesthetic attention prior to surgery), the operating theatre (where the actual surgery is performed), the ‘anaesthetic corner’ in theatre, which is the area in theatre predominantly used by anaesthetic staff, and finally recovery. A distinction between the operating theatre and the ‘anaesthetic corner’ was made, because the observations as well as a previous research [49] showed little cross-over in actors and behaviours between these two contexts, even though they were located in the same room. The waste mapping was completed for each stream until it arrived at the waste dock for pick up by external waste contractors. The latter was defined as the project boundary due to a lack of influence over contractors external to the hospital.

The workshop activities resulted in a final large paper map (dimensions 1.90 m by 3.00 m with an additional two sheets sized approximately A1). The paper map, which used a simplified version of the notation rules outlined in Table 1, was subsequently turned into a digital map using the online platform Miro [48], following all notation rules.

The second workshop also covered a prioritisation exercise to narrow down the future project focus. However, this activity and its results are beyond the scope of this paper, which focusses on behaviour identification. Subsequent prioritisation activities will be reported separately to allow for an adequately detailed description of the research undertaken and to not distract from the methodological contribution to behaviour identification the current paper makes.

## Results

**Fig. 1:**
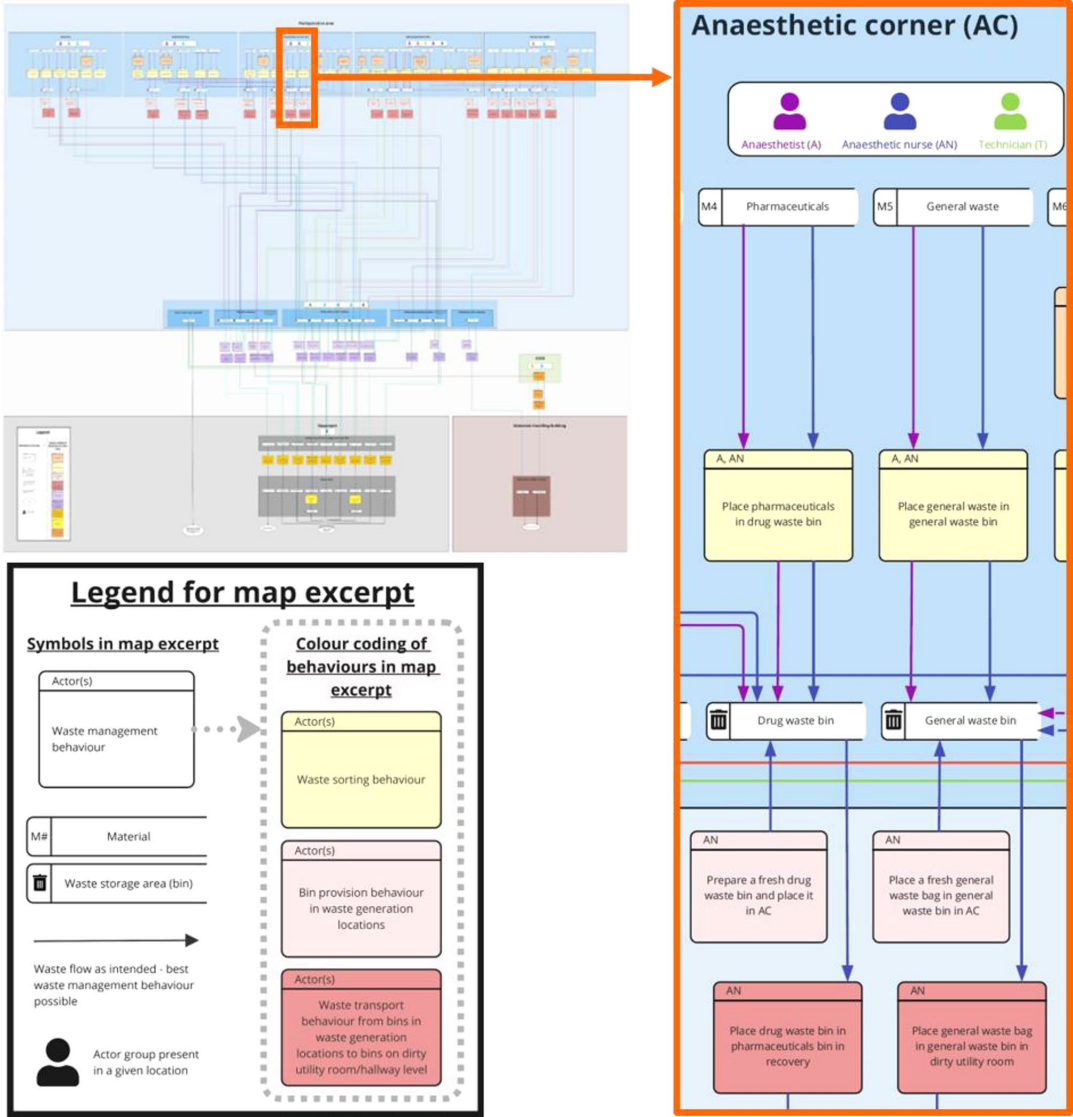
Excerpt of the waste map developed by applying the behaviour identification method

The behaviour identification method was used to develop an electronic waste map outlining all behaviours involved in generating and moving waste from the perioperative area to the waste dock (see Fig. 1 for an excerpt and Additional file 1 for the full version – please note that for space reasons the legend for Fig. 1 only includes the symbols shown in the excerpt as opposed to all symbols shown in Table 1).

The final map identified 184 waste management behaviours performed by thirteen actor groups (anaesthetists, anaesthetic nurses, recovery nurses, surgeons, surgical nurses, two types of technicians, night-time cleaning staff, day-time cleaning staff, waste team, a specific staff member of facilities management, an environmental champion and central sterile services department (CSSD) staff). The waste management behaviours covered 15 materials: Commingled recycling, PVC, sharps, pharmaceuticals, general waste, paper, soft plastics, single-use instruments, clinical waste, linen, diathermy leads, cytotoxic, huck towels, aluminium and sterilisation wrap.

67 of the identified behaviours were sorting behaviours (e.g. ‘In the set-up room, surgical nurses place commingled recycling in commingled recycling bin’). In preparation for some of these sorting behaviours, an additional 17 behaviours needed to occur, where materials got stored before they could be transported to their respective bin if that bin was located further away (e.g. ‘In the set-up room, surgical nurses store sterilisation wrap in interim location’).

Another 25 behaviours represented transport behaviours from bins in waste generation locations to bins on the dirty utility room or hallway level (e.g. ‘Cleaners empty commingled recycling bin - from set-up room - in commingled recycling bin in dirty utility room’), while an additional 25 behaviours represented bin provision behaviours in the waste generation locations (e.g. ‘Cleaners return commingled recycling bin in set-up room after emptying’).

19 behaviours were transport behaviours from the dirty utility room or hallway level to the next specific storage location, mostly the basement (e.g. ‘If full during the day, technicians transport full commingled recycling bin - from the dirty utility room - to basement’). Another 17 behaviours represented bin provision behaviours for the dirty utility room/hallway level (e.g. ‘Overnight, cleaners place an empty commingled recycling bin in dirty utility room’).

11 behaviours were transport behaviours from an interim storage location in the basement to the waste dock or into a compactor (e.g. ‘Waste team transport commingled recycling bins from temporary storage area near lifts to waste dock storage area’) and three were behaviours related to the logistics and cleaning of single-use instruments for recycling.

The identified behaviours showed how interconnected organisational waste management behaviours are. For a waste item in a particular stream to leave the hospital in that stream, often multiple actors and behaviours were required: The item needed to be placed in the correct bin by the first actor (e.g. a surgical nurse, an anaesthetic nurse, etc.), this bin or its contents then needed to be transported to various interim storage locations, usually involving a number of different actors (e.g. technicians, cleaners, etc.), until it arrived at the waste dock. If one behaviour in the chain was not performed as desired (e.g. if the contents of a recycling bin got put into a general waste bin during one of the transport behaviours), all previous sorting and correct transport behaviours would become meaningless. Mapping the waste management process as done in the behaviour identification method allowed to account for this interconnectedness of behaviours. It furthermore offered more clarity than a mere text description would [43].

## Discussion

The research described in this paper is the first known healthcare waste mapping method that incorporates behavioural insights. The case study applying the developed behaviour identification method identified 184 behaviours involved in the management of waste originating from a perioperative area in a large, metropolitan, public hospital in Australia. The behaviour identification method has three key strengths: (1) It allows for a *comprehensive* behaviour identification; (2) It ensures that behaviours are articulated to the level of detail stipulated by common behaviour change literature, i.e. including actor, action, target, context and time (where necessary) [25, 27]; (3) It accounts for the interconnectedness of behaviours involved in healthcare waste management.

The comprehensive identification is ensured through augmenting WFDs [42–44] with rules around the layout of the map and with the ‘material box’ depicting each material in each in-scope location as it turns into waste. Both of these aspects formed the crucial starting point of the waste map created based on the behaviour identification method. Facilitated by the layout rules, doing so allows the user to systematically map the ‘vertical’ journey from waste generation in one stream to the final destination of this stream in the waste system. Doing this for each ‘horizontally’ listed waste material that is created (facilitated by the introduction of the ‘material box’), the method ensures that each waste management step for each material is included.

Describing each step in behavioural terms, which is achieved by refining the ‘process boxes’ to explicit ‘behaviour boxes’ (requiring a *detailed* description of each behaviour), finally allows for the comprehensive identification of all involved behaviours.

By visually mapping the journey of each material through the waste system, and connecting related steps with arrows, the interconnectedness of all involved behaviours is represented. This visual mapping approach aligns with recent developments in the behaviour change field, which have started to create ‘behavioural systems maps’ [33, 50, 51]. These maps combine approaches from behavioural science and systems thinking and depict the interconnectedness between different actors, behaviours and influential factors on behaviours with regards to a given topic [50]. Behavioural systems mapping has been designed to investigate complex problems, such as decarbonising existing housing stock in the UK [50] or understanding the waste management of a whole region [52]. However, given that waste management behaviours in a specific healthcare setting occur in a much more linear fashion (i.e. from generation to the first bin, to the next bin, etc.) than it is the case in abstract, complex problems, it was deemed more appropriate to draw on an adaptation of WFDs [42–44] than on behavioural systems mapping for this research.

Regarding the practical implications of this research, the behaviour identification method developed in this study can serve anyone interested in designing a behaviour change intervention to improve healthcare waste management in a given local context. It addresses a fundamental step required in behaviour change projects: The identification of behaviours required to address the problem at hand [19, 29]. A waste map created based on this method can be used by clinical or environmental services staff, healthcare sustainability managers or researchers interested in collating all behaviours involved in the healthcare waste management at a given healthcare site. In line with behavioural science, the developed method deliberately expresses all behaviours as desirable options, rather than depicting which undesirable behaviours are currently occurring [24, 29]. Doing so means that users, in a subsequent step, need to review which of the identified desirable behaviours are already performed well and which have room for improvement. This activity can reduce the number of behaviours to those which require a behaviour change intervention for actors to perform the identified desirable behaviours instead of any competing undesirable behaviours. Such a prioritisation exercise avoids focussing any subsequent efforts on behaviours which are already working well. Given that clinical staff often are time-poor [53], a pragmatic way to support such a prioritisation can be a ‘traffic-light’ exercise, where behaviours that are performed well get marked green, while behaviours, which are not working, get marked yellow or red, depending on the severity of the issues. As an optional addition, the prioritisation exercise can be augmented by explicitly noting which competing undesirable behaviours are being performed instead of any desirable behaviours. Such an addition fosters an even deeper understanding of the local context.

Once the most relevant desirable behaviours for a given project have been selected, facilitators and barriers to behaviour performance can be investigated. Subsequently, a targeted intervention to reduce identified barriers and strengthen identified facilitators can be designed, thereby increasing the likelihood of success for a given intervention [19].

As many studies, this study does not come without limitations. To ensure that time-poor clinical staff could participate in the case study workshops, not all rooms in the perioperative area of the partner hospital were included. Additionally, some smaller material streams were omitted in the waste map, such as batteries or confidential paper waste. More time, or an initial waste audit to identify all created materials, could have improved the created map in this regard. Thus, while the created waste map in this case study did not comprehensively identify every single behaviour involved in managing waste from the perioperative area of the partner hospital, the behaviour identification method itself still allows to do so, as long as the included rooms and materials are comprehensively represented in the map.

Furthermore, as can be seen in the map, a number of identified behaviours omitted the behavioural component of ‘time’. This was deemed necessary to make the most efficient use of the available workshop time. Explicitly adding in the time element to every identified behaviour would have required longer workshop sessions, which would have been challenging for participants to commit to. However, given that the identified behaviours describe the waste disposal process *as intended*, it is reasonable to assume that the time element can simply be defined as ‘always’ unless specified otherwise.

The case study described in this study identified desirable waste management behaviours in the partner hospital based on observations and participants’ insights at the time of the workshops. Future research could compare the perspectives of workshop participants with various existing guidelines that describe waste management. It should also be noted, that while the developed *method* to identify waste management behaviours in a healthcare setting is expected to be applicable to other healthcare settings, the 184 waste management behaviours identified in this study, are specific to the partner hospital of this research and are highly dependent on the local context of this hospital. It is anticipated that using the developed method in a different healthcare site will identify different waste management behaviours to the current study. Hence, future research could further explore the applicability of the method in other hospital and healthcare settings, beyond the perioperative area tested in this study. Additionally, while the behaviour identification method was developed to identify waste management behaviours in a healthcare setting, it can be used to identify waste management behaviours in any organisational setting, as this typically involves multiple staff groups and interconnected waste management behaviours. Further research could investigate the usefulness of the method in other organisational settings, such as offices. Finally, it has been suggested that WFDs can support staff training, are beneficial for intervention design and allowed to identify potential cost savings of UK £ 28,099 in one hospital [42, 43]. Thus, the behaviour identification method’s usefulness beyond the mere identification of behaviours in waste management projects should be tested in future research.

## Conclusion

This research developed and tested a method to comprehensively identify and map the often interconnected waste management behaviours in a healthcare setting. To do so, previous work on WFDs [42–44] was advanced by overlaying it with principles of behavioural science, clearly defining the behaviours involved in the waste management process. Applying the developed method to the management of waste from a perioperative area in a large, metropolitan, public hospital in Australia identified 184 interconnected waste management behaviours, performed by thirteen actor groups.

The developed behaviour identification method lays the foundation for behaviour prioritisation and intervention design by enabling the identification of a comprehensive pool of behaviours to prioritise from. Ensuring that all potential target behaviours are identified, reduces the risk of missing important behaviours. Furthermore, the visualisation in a map ensures that interconnected behaviours are not considered in isolation, which supports an understanding of subsequent behaviour change efforts in the context of an interconnected waste management system. The developed behaviour identification method is readily adaptable to other waste management settings.

## Supplementary Information

Below is the link to the electronic supplementary material.

Supplementary Material 1

## Data Availability

Data that support the findings of this study cannot be shared because the obtained ethics approval does not cover the sharing of raw data.
