# Supplementary figures and images for: Developing a behaviour identification method for healthcare waste management: a case study of hospital waste management in Australia
Source: BMC Health Serv Res. 2025 Oct 2;25:1288. doi: 10.1186/s12913-025-13463-5 (PMC12492881; doi:10.1186/s12913-025-13463-5)

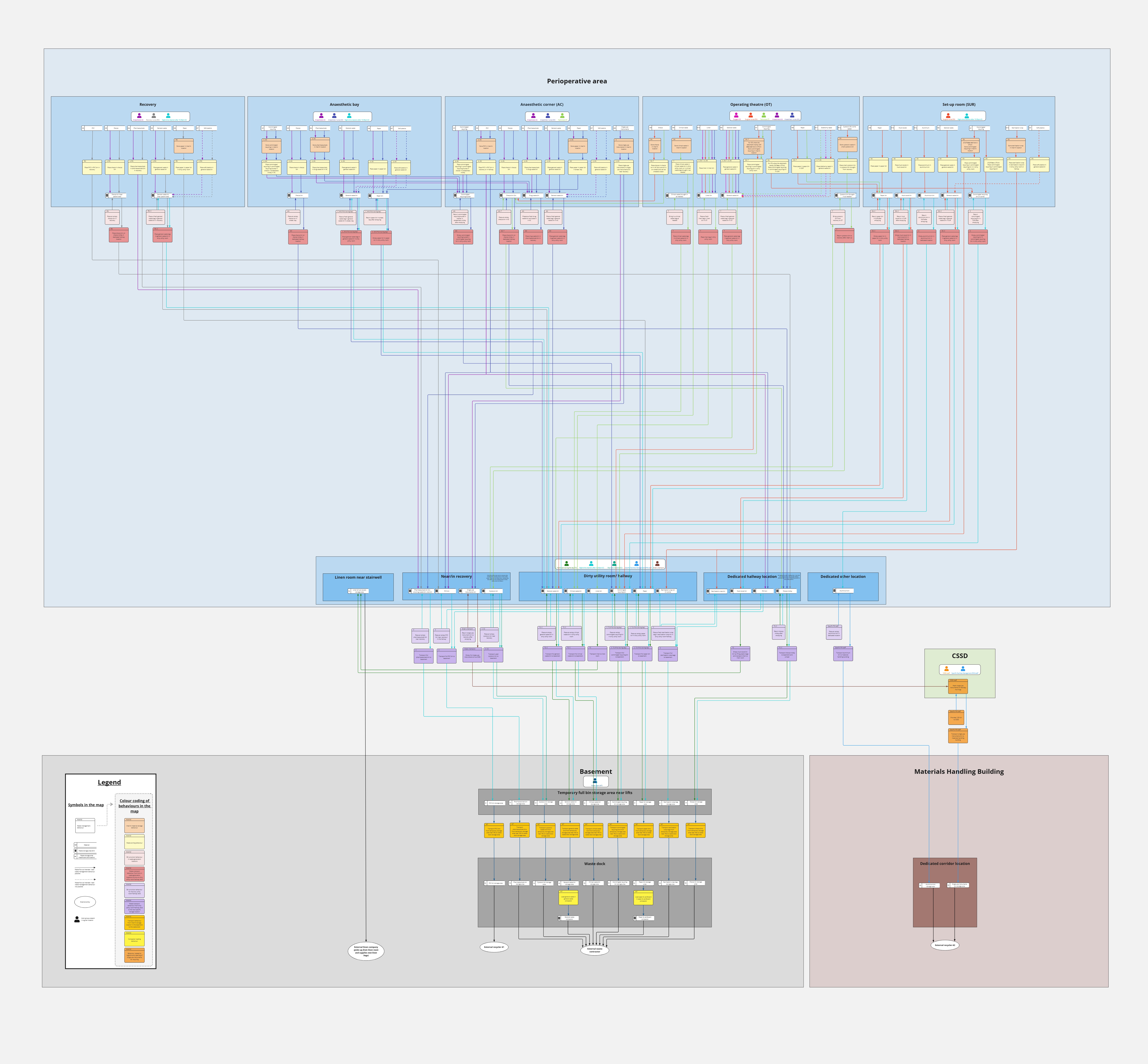

Supplement: Supplementary file 1 — Supplementary Material 1 [file 12913_2025_13463_MOESM1_ESM.jpg]
